# Electronegativity Equilibration

**DOI:** 10.1021/acs.jpca.2c03814

**Published:** 2022-08-08

**Authors:** Francesco Sessa, Martin Rahm

**Affiliations:** Department of Chemistry and Chemical Engineering, Chalmers University of Technology, SE-412 96 Gothenburg, Sweden

## Abstract

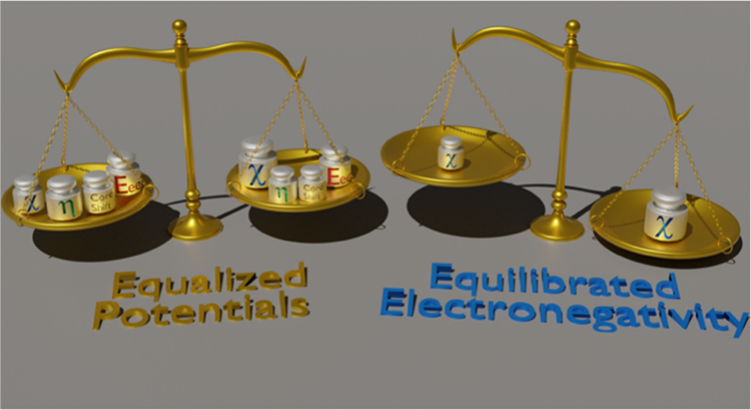

Controlling the distribution of electrons in materials is the holy grail of chemistry and material science. Practical attempts at this feat are common but are often reliant on simplistic arguments based on electronegativity. One challenge is knowing when such arguments work, and which other factors may play a role. Ultimately, electrons move to equalize chemical potentials. In this work, we outline a theory in which chemical potentials of atoms and molecules are expressed in terms of reinterpretations of common chemical concepts and some physical quantities: electronegativity, chemical hardness, and the sensitivity of electronic repulsion and core levels with respect to changes in the electron density. At the zero-temperature limit, an expression of the Fermi level emerges that helps to connect several of these quantities to a plethora of material properties, theories and phenomena predominantly explored in condensed matter physics. Our theory runs counter to Sanderson’s postulate of electronegativity equalization and allows a perspective in which electronegativities of bonded atoms need not be equal. As chemical potentials equalize in this framework, electronegativities equilibrate.

## Introduction

The goal of this work is to open new possibilities for rationalizing charge transfer, by practically connecting the chemical potential to reinterpretations of well-established chemical and physical concepts. To do so, we describe a theory that explicitly links one set of definitions of electronegativity and chemical hardness to the chemical potential and the Fermi energy of molecular and condensed phase systems. Detailed definitions of these concepts and other factors will be introduced gradually during the discussion.

Since Pauling formalized the idea in 1932,^1^ electronegativity has grown into a useful tool for rationalizing trends in charge transfer, bond polarity, bond strength, reactivity, and various chemical properties. Electronegativity is a simple and intuitive concept in that it quantifies the ability of atoms (or groups of atoms) to attract (or hold on to) electrons. Over time, many different electronegativity scales have been proposed (e.g., refs (1−20)), each attempting to tie this chemical concept to different physical properties, typically including ionization energies, electron affinities and other spectroscopical properties of valence electrons. Some electronegativity scales are based on physical properties of bonded atoms, such as the Pauling or Walsh scales,^1,6^ while other are defined from properties of isolated atoms, such as the Mulliken, Allred & Rochow and Allen scales.^8,13,21^ A particular challenge with electronegativity is to reconcile its many definitions with its common practice and resulting chemical predictions (and failed predictions). How electronegativity changes upon bond formation is, consequently, a long debated topic.^4,7,14,22−47^

A well-known postulate by Sanderson states that electronegativity of atoms in molecules should equal the mean of the values for the isolated atoms.^7,10^ In other words, the electronegativity of isolated atoms is postulated to equalize when atoms are bonded together inside molecules or materials. Sanderson’s idea was supported by Parr and co-workers, who defined electronegativity as the negative of the chemical potential.^48^ In turn, the chemical potential is a homogeneous value across any system in equilibrium. Chemical potentials determine where electrons flow.^49^ Nevertheless, alternative viewpoints on electronegativity equalization have been voiced by several.^4,14,44−47,50^ One of the critiques to Sanderson’s postulate is rooted in the chemical expectation that electronegativity should reflect how the nature of atoms differs, even when they are bonded in a material. Equalization requires all atoms to adopt the same electronegativity within a molecule, regardless of the element. A related objection is that Sanderson’s postulate necessitates electronegativity to be defined as the chemical potential.^44,48^ In other words, Sanderson’s postulate excludes a host of electronegativity definitions, many of which are by their own right useful. Many opposing viewpoints on electronegativity have merit but it is outside our scope to weigh them. We stress that this work is not intended as a critique of related theories and methods that can provide chemical insight (e.g., refs (51−63)). Rather, we aim to develop a complementary perspective.

Here, we rely on an established definition of electronegativity that is connected to the average electron energy (χ̅)^64−66^
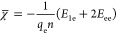
1where *E*_1e_ and *E*_ee_ are the one- and multi-electron energies of a system, respectively, *n* is the total number of electrons and *q*_e_ is the elementary charge. The average electron energy can be formally partitioned so that
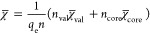
2and electronegativity attributed to the average valence electron energy, χ̅_val_. This definition is inspired by, but not equal to, that of Allen^13^ and has allowed for the compilation of an extensive ground state (*T* → 0 K) scale of the electronegativity of atoms.^64^ This scale has been productively used in literature, for example to rationalize trends in intermetallic compounds.^67−70^ It has also been extended to high pressure conditions,^64,71−73^ allowing for the successful rationalization of unexpected phenomena, such as redox inversion in nitric sulfur hydrides.^74^

How to formally separate core from valence in eq 2 is not always obvious. However, such choices do not influence the validity of our following arguments and results. We refer to previous work for a detailed discussion on what constitutes a valence level,^64^ and for a comparison with the Allen electronegativity scale.^13^ We note that both Allen’s and our preferred scale of electronegativity are related to the average local ionization energy that has been extensively studied by Politzer and co-workers.^18^ The average electron energy is also related to the theory of moments of the electronic energy distribution, useful for rationalizing structure in extended systems.^75−78^

Our choice for defining electronegativity allows us to connect this central chemical concept to the total energy of a system^65,66^
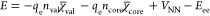
3

In eq 3*V*_NN_ and *E*_ee_ are the electrostatic repulsions between nuclei and electrons, respectively. Equation 3 is exact within the Born–Oppenheimer approximation, and all its terms can be evaluated quantum-mechanically, at various levels of theory, as well as be estimated experimentally through a combination of techniques.^64−66^ For example, the value of χ̅_val_ can be experimentally estimated as an average of photoionization energies of valence levels,^64^ or be approximated by averaging the energy of occupied valence orbitals. Because of the possibility of interchangeable use of both experimental and computed data, we refer to eq 3 as an experimental quantum chemistry (EQC) energy decomposition scheme.^65,66^ There is no unique way to decompose energies and several other elegant methods exist to do so (e.g., see refs (79−102)). EQC energy terms have been successfully used to, for example, classify chemical bonds in diatomic molecules^65,66^ and small hydroxides,^103^ for distinguishing red- and blue-shifted hydrogen bonds,^104^ and for estimating substituent effects in benzoic acids.^105^

In what follows, we develop upon the EQC framework to address its relationship with the chemical potential. As we do so, we will prove why, with our definition, electronegativity equilibrates within molecules rather than equalizes.^51−63^ We begin by exploring an expression of the chemical potential that connects to the notion of electronegativity in a new way.

## Methods

All density functional theory (DFT) calculations were performed at the LC-BLYP/TZP level of theory, using ADF version 2021.207.^106^ The derivative terms discussed in this work have been evaluated through a finite-difference approximation, which relies on the computation of a fractional occupation number of the highest occupied molecular orbital (HOMO). The addition and removal of non-integer numbers of electrons (±0.01 e^–^ in this work) is a mathematical trick allowed by DFT. This approach can be regarded as a small perturbation to the electronic structure. Alternatively, in a statistical perspective, fractional HOMO occupations approximate the ionization of a small percentage of systems within an ensemble.^107^ The range-separated LC-BLYP functional was chosen as it appears nearly self-interaction-error free, producing energies that are linear as a function of fractional charge for the investigated systems.^108^ Even systems without an electron affinity can be studied provided that the basis set is sufficiently large. For example, the energy of the HOMO of N^–^ calculates as negative, if it is evaluated from the ∂*E*/∂*N* derivative using the LC-BLYP functional.^108^ Whereas any DFT functional is approximate, trends and conclusions of this work are not sensitive to the level of theory. All experimental values of atomic polarizabilities are from the CRC Handbook of Chemistry and Physics.^109^

## Results and Discussion

### Chemical Potential and Electronegativity

The chemical potential, μ, quantifies the rate of change in the Gibbs free energy *G* when an ensemble of systems exchanges particles (e.g., molecules or electrons) with a surrounding environment
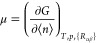
4

**Figure 1:**
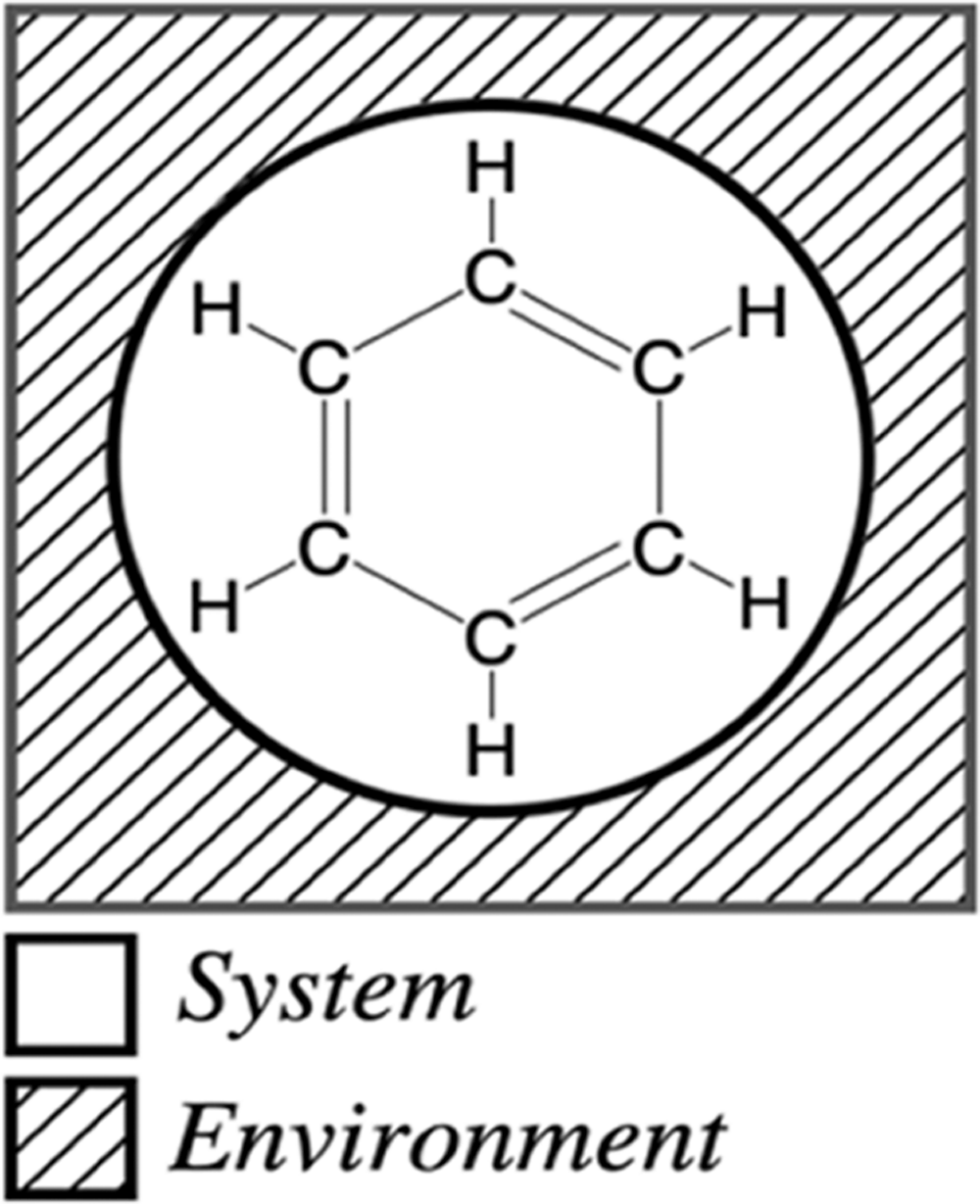
Chemical potential is defined for an *n*-electron system exchanging electrons with a surrounding environment. The environment acts as an infinite electron reservoir.

In eq 4, *n* is the number of particles, *T* is the temperature, *p* is the pressure and {*R*_αβ_} are the internal coordinates that define the geometry. The subscript in eq 4 indicates that we consider *G* as being differentiated at constant temperature, pressure and system geometry. The symbol ⟨...⟩ represents ensemble averaging. The theory to be described will consider a grand canonical (μ*VT*) ensemble.^49^ Equilibrium in such an ensemble means that each system has reached the same chemical potential, temperature and volume. In other words, the free energy is at a minimum. Figure 1 illustrates an example of an *n*-electron system surrounded by an environment that acts as an infinite electron reservoir with which the system can exchange electrons. A large collection of such open systems, all in equilibrium with a reservoir, is by definition a grand canonical ensemble.^49^

What is the connection between eq 4 and electronegativity? With the help of eq 3, the Gibbs free energy can be expressed within EQC as
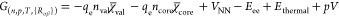
5where *V* is volume and *E*_thermal_ includes all temperature and entropy contributions. By substituting eqs 5 into 4, we obtain an explicit expression for the chemical potential as a function of electronegativity
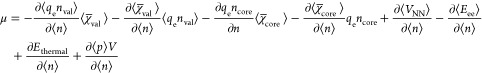
6

Equation 6 looks complicated but can be simplified. Since the partial derivatives in eq 6 are considered at constant geometry, . Furthermore, we can assume that a system shares electrons with its environment exclusively via valence levels. The population of core levels is then a constant in the ensemble. We note that this is a chemically motivated definition of what constitutes a core level: a level that cannot, in chemical interactions, share electrons with a surrounding environment. From such a definition follows that . We can then rewrite eq 6
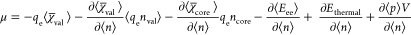
7

Equation 7 expresses the temperature and pressure dependence of the chemical potential and its relationship with electronegativity. An arguably relevant case is the in vacuo zero-temperature limit of μ, which corresponds to the Fermi energy (ε_F_) of a system^49^
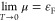
8

In vacuum and at *T* → 0 K, we have no pressure contribution, i.e., , and the thermal contribution is negligible, . Equation 7 then simplifies further
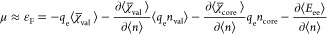
9

Equation 9 defines the Fermi energy within the EQC framework. Several factors are revealed to influence the Fermi energy, the first of which is our definition of electronegativity, ⟨χ̅_val_⟩. The second term of eq 9, which includes the derivative , describes how the electronegativity (the average energy of valence electrons) of a system changes as a function of its electronic population. The larger this derivative is, the larger impact does a change in the electron density have on the electronic potential of that system. The  term will be important when we return to discuss chemical hardness in the context of EQC. The third term, , is related to the shift of core levels upon ionization of a system. Core electrons arguably have no direct role in chemical bonding. However, this third term formalizes how the movement of core levels relate to the chemical potential, which, in turn, can change over any transformation. The last term of eq 9, , describes the electron repulsion response to the system gaining or losing electrons.

Table 1 shows a test of eq 9 in which we have calculated μ and each of its components for all atoms of the first three rows of the periodic table. In these examples, χ̅ is approximated as the average energy of occupied Kohn–Sham (KS) orbitals, while the derivatives in eq 9 are evaluated through a finite-difference approximation (see the Methods section). The results of eq 9 agree nearly perfectly with the in-practice definition of the Fermi level in computational physics and chemistry: the average energy of the HOMO and the lowest unoccupied (LUMO) orbitals. This agreement is significant because the terms of eq 9 are all evaluated from occupied electronic states, avoiding the use of virtual orbitals. The root mean square deviation between these two options for estimating μ, or ε_F_, is 0.06 eV.

**Table 1:** Components of the Chemical Potential μ, Evaluated for Atoms in the First Three Rows of the Periodic Table^a^

element	⟨χ̅_val_⟩(eV·e^–1^)	(eV·e^–1^)	(eV·e^–1^)	(eV)	μ (eV)	ε_F_ (eV)
H	12.06	–12.62	0.00	7.10	–6.53	–6.43
He	21.89	–21.02	0.00	26.33	–6.19	–6.13
Li	5.38	–5.10	–7.01	16.76	–3.02	–2.97
Be	8.72	–8.27	–9.56	30.77	–3.82	–3.78
B	11.58	–9.93	–10.79	43.91	–4.11	–4.09
C	14.47	–11.70	–12.99	64.24	–5.94	–5.96
N	17.48	–13.51	–14.26	85.29	–6.71	–6.69
O	20.07	–15.18	–16.02	110.56	–7.48	–7.30
F	22.84	–16.70	–17.96	140.18	–10.24	–10.15
Ne	25.82	–16.38	–16.07	142.14	–4.75	–4.76
Na	5.16	–4.83	–6.43	66.85	–2.91	–2.87
Mg	7.38	–7.25	–8.02	90.05	–2.72	–2.69
Al	9.30	–7.24	–7.18	87.17	–2.95	–2.96
Si	11.30	–8.10	–8.53	110.90	–4.48	–4.47
P	13.40	–9.04	–9.37	130.95	–5.41	–5.40
S	15.16	–10.11	–10.40	155.59	–6.12	–6.11
Cl	17.06	–10.96	–11.72	185.05	–8.19	–8.15
Ar	19.12	–10.84	–10.61	177.38	–3.63	–3.64

a Values of μ are calculated via eq 9. Shown for comparison is the Fermi energy ε_F_, evaluated as the average energy of the highest occupied and lowest unoccupied orbitals.

Note how the components of μ follow clear trends across rows and down groups of the periodic table. The  and  terms show surprisingly comparable magnitudes when evaluated for both second and third row atoms. Comparable energy shifts in the average valence and core levels upon partial ionization imply that core levels play a substantial (albeit indirect) role in the energetics of chemical processes. That the  and  terms are so similar in magnitude is interesting considering the successes of common pseudopotential-based calculations.^110^ The implicit approximation in such calculations is that .

### A Different Definition of Chemical Hardness

The derivative  in eq 9 reminds us of another textbook concept in chemistry: chemical hardness.^111−114^ This concept was proposed by Pearson in 1963 as an integral part of his hard and soft acids and bases (HSAB) theory.^115^ Together with Parr, Pearson later gave a quantitative definition, which became part of the conceptual density functional theory (CDFT) framework.^111^ Within CDFT, chemical hardness is defined as the negative of the derivative of electronegativity with respect to electron population.^112−114^ Since then, HSAB theory has been proven a useful tool in several chemical applications.^116−121^ What is the physical interpretation of chemical hardness?

**Figure 2:**
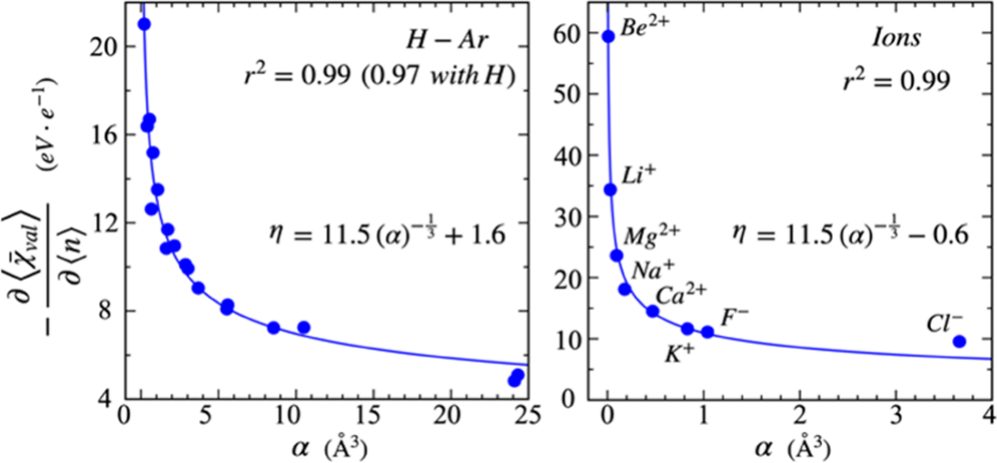
term calculated for H-Ar (left) and for a selection of ions from group 1, 2, and 17 (right), plotted against experimental polarizabilities, α.^109^

In the HSAB theory and CDFT, the hardness of a system, be it an atom, a molecule or an ion, has a well-established inverse cube root relationship to polarizability.^122,123^Figure 2 demonstrates that the exact same proportionality holds true between atomic polarizability and the negative of the  term in eq 9. The correlation is remarkably strong across atoms and a selection of alkali and alkaline-earth cations and halide anions (*r*^2^ = 0.99).

The relationship shown in Figure 2 motivates us to define chemical hardness (within the EQC framework) as
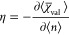
10

Because this definition of chemical hardness shows the same proportionality to polarizability as the Parr–Pearson chemical hardness,^122,123^ the two definitions are indistinguishable on a practical level. On a conceptual level, chemical hardness now shares a common definition in both EQC and CDFT, i.e., as the negative derivative of electronegativity. There is a close analogy in the physical meaning of the two definitions: in both cases hardness represents a resistance of the chemical potential to changes in the number of electrons.^112−114^ One fundamental difference is that, in CDFT, hardness is also the derivative of the chemical potential. As we will show next, analogies between the two frameworks, EQC and CDFT, go beyond the concept of chemical hardness.

### Comparing EQC with Conceptual DFT

To appreciate the analogies that follow, it is helpful to remind of some aspects of CDFT. In CDFT, electronegativity is denoted by χ and is defined as the negative of the chemical potential^48^
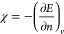
11where *E* and *n* are the DFT energy and number of electrons, respectively, and *v* is the external potential that uniquely defines the electron density of a system. The electronegativity scale defined by eq 11 was first proposed by Iczkowski and Margrave^9^ and implies Sanderson’s equalization. Because the chemical potential is homogenous throughout a system at equilibrium, all atoms in such a system will, by the CDFT definition, have equal electronegativity. A well-known approximation to this electronegativity is the Mulliken scale, χ_M_, which can be obtained by a finite-difference approach. The Mulliken electronegativity can be expressed in DFT terms as^48^
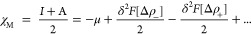
12

In eq 12*I*, *A*, μ, and *F* are the ionization potential, electron affinity, chemical potential and the universal DFT functional, respectively. Δρ_–_ and Δρ_+_ represent changes in electron density when losing or gaining an electron, respectively. Higher order terms in eq 12, and following equations in this section, are not relevant for the discussion and will be omitted. Note that eq 12 expresses χ_M_ as a function of the chemical potential. CDFT hardness also has a well-known approximation (the Parr–Pearson hardness, η_P_),^48^ obtainable by finite differences^48^
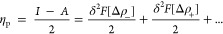
13

By combining eqs 12 and 13, and with some rearrangement, we can express the chemical potential as a function of the Mulliken electronegativity and Parr–Pearson hardness
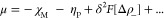
14

Equation 14 is an alternative expression for the CDFT chemical potential. However, we stress that χ_M_ and η_P_ are only approximations to the CDFT electronegativity and chemical hardness. With this consideration in mind, we can point to term-by-term analogies between our EQC-derived eq 9 and the CDFT-derived eq 14. In both equations, the first term corresponds to electronegativity and the second term relates to chemical hardness (although in eq 9, the second term also contains the number of valence electrons). The last term of both equations relates to changes in the electron-electron interactions: one of the principal components of the universal functional F[ρ] is the electron repulsion energy, as stated by Parr.^48^ We stress that these analogies should only be considered qualitative in nature. Because the definition of electronegativity and hardness differs between the EQC and CDFT frameworks, there is no formal correspondence between the right-hand side terms of eqs 9 and 14. However, the chemical potential links the two equations, and we note that both definitions of electronegativity and hardness provide values of comparable magnitude (see Table 1 and ref (124)). One noticeable difference between the EQC-derived chemical potential, eq 9, and the CDFT-derived chemical potential, eq 14, is that the former includes a term accounting explicitly for the role of core electrons.

We emphasize that our comparison with CDFT is not meant as a critique of the latter, but the contrary. By building bridges between theoretical frameworks, and highlighting commonality and complementarity of different perspectives, we become better equipped to analyze and understand electronic structure and chemical transformations. A notable merit of the CDFT framework is the establishment of a series of electronic structure principles connected to reactivity, such as the “|Δμ| big is good” rule^125,126^ and the principle of maximum hardness.^127,128^ A detailed discussion of these principles within the EQC framework is beyond the scope of this work. However, we note that any principle based on the chemical potential will remain valid within EQC, even as a given value may find a complementary interpretation through eqs 7 or 9. The principle of maximum hardness is likely to remain valid in EQC. We postulate this as both the EQC and CDFT definitions of chemical hardness result in strong connections to polarizability, and because the response to electronic perturbations is invariably linked to the HOMO energy.

With a new expression for the chemical potential as a function of electronegativity in hand, we can proceed to discuss the equilibria that come into play when atoms join to form bonds. How does our definition of electronegativity of atoms change in a molecular environment?

### Atomic Electronegativity and the Chemical Potential

**Figure 3:**
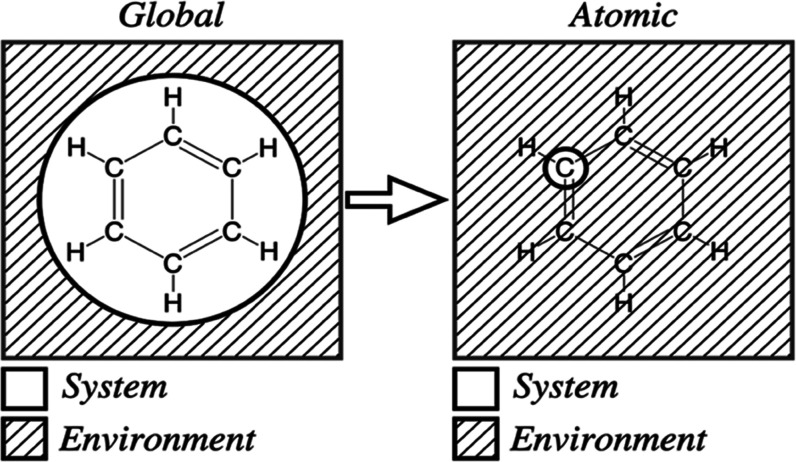
Molecular, or global, quantities are defined for an ensemble of systems exchanging electrons with the environment (left panel). Atomic quantities can be defined similarly, as an ensemble of single atoms, while the rest of the system becomes part of the environment (right panel).

Moving away from global quantities that describe a system as a whole, we next study atomic properties. As we do so, we change our point of view to consider each atom within a molecule, crystal, or any material, as a sub-system. Figure 3 illustrates this change in perspective.

For each atomic sub-system, the rest of the molecule can be treated as the environment with which the atom exchanges electrons. In this perspective, each atom is represented by a grand canonical ensemble in chemical and thermal contact with the ensembles representing the other bonded atoms. Upon equilibration, the chemical potential of such atomic ensembles will be equal, which allows their combination into a larger molecular ensemble, i.e., the general case we discussed previously.

To consider molecules as comprising of different fragments is common practice in chemistry. However, there is no unique way to quantum mechanically do so. In principle, the framework we propose herein can make use of any partitioning method, regardless of whether they are, for example, based on topological analysis of electron densities,^129,130^ or population analysis of wavefunctions.^131,132^ By partitioning into atoms, we can recast the Gibbs free energy expression of a molecule, eq 5, as a function of atomic electronegativities
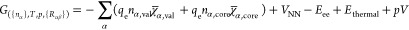
15where the index α runs over all atoms in a molecule. We can then redefine the chemical potential of an atom that is exchanging electrons with the rest of the molecule, our system and environment, respectively
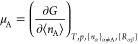
16

In eq 16, *n*_A_ is the electronic population of an atom A, and the subscript {*n*_α_}_α≠A_ means that *G* is differentiated at constant populations for all atoms except A. Note that this atomic chemical potential has the same functional form as the macroscopic chemical potential of a component in a multi-component mixture.^133^ However, here *n*_A_ is the number of electrons attributed to atom A, while in a macroscopic system *n*_A_ would represent the number of particles of component A in a mixture. Following the same reasoning illustrated before for an ensemble of *n*-electron molecular systems (see eq 6), we can express the atomic chemical potential as
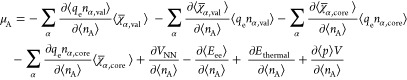
17

If we maintain that exchanges of electrons only proceed via valence levels (vide supra), we have  and , where δ_αA_ is either 0 or 1 depending on whether α ≠ A or α = A, respectively. We also know that . Finally, for the in vacuo zero-temperature limit of μ_A_ the terms  and  are negligible. We can then simplify eq 17
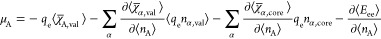
18

Equation 18 shows how the chemical potential of an atom A, in a molecular environment, is a function of its electronegativity. Note that the label A marks the specific atom for which the chemical potential μ_A_ is evaluated, while the index α runs over all atoms in the molecule. At face value, eq 18 looks similar to eq 9. However, the latter describes the properties of the whole system, with no external forces influencing its chemical potential. In contrast, eq 18 makes explicit the connection between the electronegativity and the chemical potential of an atom within a molecular environment. Expressing chemical potentials of atoms as in eq 18 provides a perspective closely related to the well-known example of a junction between p- and n-type semiconductors.^49^ When two different semiconductors are put in contact, electrons flow from a higher chemical potential to a lower one. In a p–n junction, the chemical potential is homogeneous at equilibrium. However, each side of the junction is still expressed as a sum of two inhomogeneous quantities. One is a local potential, which relates to the type of the semiconductor. The second is an external electrostatic potential, which arises from inhomogeneous doping of the two semiconductors.^49^ We argue that the flow of electrons between bonded atoms can be described in similar terms, by the chemical potentials provided by eq 18.

With expressions for atomic chemical potential in hand, it is possible to next address the electronegativity equilibration that takes place upon chemical bond formation.

### Electronegativity Equilibration

When bonds are formed, electron density is redistributed among atoms as to minimize the Gibbs energy, i.e., d*G* = 0. Equation 15 shows the Gibbs energy of a system as a function of temperature, pressure, atomic populations, and its geometry. If we consider a bonding process at constant temperature and pressure, the total differential of that energy can be expressed as
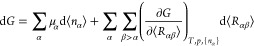
19

Borrowing a perspective from Marcus theory,^134^ the change in populations—at fixed geometry—is the proper reaction coordinate of an electron transfer (ET) process. The first term on the right-hand side of eq 19 corresponds to the change in energy along this ideal ET reaction coordinate. Conversely, the last term of eq 19 captures the response of Gibbs free energy to changes in nuclear geometry. In DFT terminology, this last term is the energy response due to a change in the external potential. The different  terms relate directly to Hellman-Feynman forces, i.e., the forces experienced by nuclei in a molecule.^135,136^ We note that Averill and Painter have studied and named similar terms “dynamic orbital forces”.^136^

In a similar fashion to eq 18, we next outline an EQC expression of  for any given pair of atoms
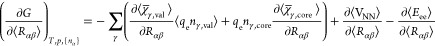
20where α and β denote a specific pair of atoms, while γ runs over all atoms. Equation 20 tells us that the forces experienced by nuclei are mainly influenced by two factors: (1) how energies of electronic levels shift because of nuclear movement, and (2) the changing balance between electrostatic repulsions (nuclear-nuclear and electron-electron).

By substituting eqs 18 and 20 into eq 19, and with some rearrangements, we can express the energy differential in a chemical process in terms of atomic electronegativities
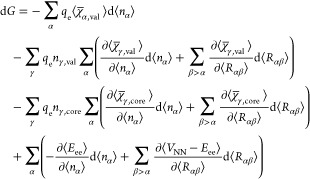
21where α, β and γ run over all atoms. Note how the chemical potential of *each* atom contains sums over all atoms when expressed in eq 18. Summing over the chemical potential for all atoms in eq 19 consequently results in a double summation in eq 21. Similarly, the sum over all atoms in eq 20 results into the triple summations in eq 21.

By converting the sums over all the partial derivatives into total differentials we can write a more compact expression for eq 21
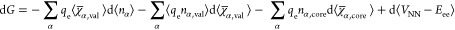
22

Equation 22 describes energy changes in any chemical process. For example, when integrated over a chemical reaction eq 22 allows the study of how individual atomic energy contributions evolve. We focus on the conceptual meaning of the first right-hand side term of eq 22 in what follows but remind that the second term relates to chemical hardness. The quantification of all these terms is possible through different methods (e.g., see ref (137)) and we will return to discuss their physical rationale and implications in detail, in future work.

When d*G* equals zero, eq 22 represents the equilibrium condition between atoms exchanging electrons within a molecule, or any other material. In such a case, the first right-hand side term of eq 22 is proportional to an electronegativity difference. To see why, we look at the example of a heteronuclear diatomic molecule AB. In such a molecule, the change in electron population of one atom (relative to the isolated atom) must perfectly equal that of the other atom, but with reversed sign, i.e., d⟨*n*_A_⟩ = −d⟨*n*_B_⟩. It then follows that ∑_α_*q*_e_⟨χ̅_α,val_⟩d⟨*n*_α_⟩ is equal to a change in electronic population times the difference in electronegativity between the two atoms in the molecule, d⟨*n*_α_⟩(⟨χ̅_B,val_⟩ – ⟨χ̅_A,val_⟩).

Equation 22 explains why differences in electronegativity between non-equivalent atoms (or fragments) are expected to persist at equilibrium. Atomic electronegativities (of the sort we investigate cf., eqs 1 and 2) can equalize only in special cases where contributions from hardness, core shifts and electrostatic repulsions cancel out. Equation 22 therefore formalizes the concept of electronegativity equilibration and explains why equalization of electronegativity is not strictly necessary.

## Conclusions

Independently of its precise definition, electronegativity is a powerful concept for quickly predicting the direction of charge transfer and bond polarity. But even so, we know that trends in this atomic property can disagree with a range of observables, such as trends in bond energies, reactivity or stability. For example, the dipole moments in molecules such as CO, CS and BF are inverted relative to expectations from simple electronegativity arguments (these dipole moments can be explained in other ways).^138−140^ We believe this work constitutes an important step towards better understanding the occasional practical shortcomings of electronegativity.

Chemical potentials are what ultimately determines where electrons move. In the theory we present, the chemical potential is expressed in terms of reinterpretations of several well-known chemical concepts and physical quantities, including electronegativity, chemical hardness, changes in electronic repulsions within a molecule, and the sensitivity of core levels to changes in the electron density. One effect formalized (but not explored in this work) is the effect of pressure on the chemical potential. Conditions of high pressure are likely to significantly affect equilibration of electronegativity in chemical reactions, as also suggested elsewhere.^71,73^ At the zero-temperature limit, an expression of the Fermi level emerges that, we think, helps to connect several central chemical concepts to a plethora of material properties, theories and phenomena predominantly explored in condensed matter physics.

In this work, we make clear that with a definition of electronegativity as the average energy of valence electrons (a definition related but not equal to that of Allen^13,64^) we allow for a perspective in which atoms within molecules and materials can have different electronegativities. This premise is at odds with the electronegativity equalization postulate of Sanderson, which holds true only when electronegativity is defined as the negative of the chemical potential. Our analysis is enabled by the theoretical framework EQC, which is intended to encompass and connect useful concepts within theoretical chemistry and physics. Analogies and differences between EQC and CDFT are discussed, and we stress the complementarity of these two approaches.

One motivation for EQC is to facilitate the interchangeable use of theoretically and experimentally obtained data when analyzing electronic structure. Several terms in our partitioning of the chemical potential, eq 9, can, in principle, be estimated experimentally. Valence and core levels can be probed by photoemission spectroscopies, and so should their sensitivity to electronic perturbations. For atomic quantities, a strict connection between theory and experiment becomes harder. Core energies can be probed selectively by X-ray spectroscopy in many instances. However, attributing experimental estimates of average binding energies of valence electrons to atoms inside molecules is challenging. Developments of the resonant inelastic X-ray scattering (RIXS) technique are encouraging, as it may allow for the probing of valence levels with atomic selectivity.^141−143^

We intend for the outlined theory to stimulate constructive discussion on the driving forces responsible for chemistry, and the limits of chemical rationales in chemistry at large.
